# Profile of Trace Elements in Selected Medicinal Plants Used for the Treatment of Diabetes in Eritrea

**DOI:** 10.1155/2016/2752836

**Published:** 2016-10-04

**Authors:** Mussie Sium, Patrick Kareru, Joseph Keriko, Berhane Girmay, Ghebrehiwet Medhanie, Semere Debretsion

**Affiliations:** ^1^Department of Chemistry, College of Science, Eritrea Institute of Technology, 1056 Maekel, Eritrea; ^2^Department of Chemistry, Jomo Kenyatta University of Agriculture and Technology (JKUAT), Nairobi 62000-00200, Kenya; ^3^Department of Chemistry, School of Pharmacy, College of Health Sciences, 8566 Asmara, Eritrea; ^4^SGS, Mineral Assay Laboratory, Bisha Mining Share Company, 4275 Asmara, Eritrea

## Abstract

This study was designed to investigate the profile of certain trace elements having therapeutic properties related to diabetes mellitus. The investigated plants were* Aloe camperi*,* Meriandra dianthera*,* Lepidium sativum*,* Brassica nigra, and Nigella sativa*. These plants are traditionally used in the management of diabetes in Eritrea. The elemental analysis was conducted using inductively coupled plasma optical emission spectrometry (ICP-OES) and flame atomic absorption spectroscopy (FAAS) techniques. The accuracy of the methods was verified using in-house reference materials (CRMs) and no significant differences were observed between the measured and certified values. The analysis displayed variable concentrations of the different trace elements including Zn, Cr, V, Mn, and Se in the plants. Moreover, the levels of major elements, such as Mg, Ca, K, Na, and Ba, and heavy metals, such as Fe, Cu, Ni, Co, As, and Pb, were determined and found to be in the permissible limit defined by WHO. Among the plants,* Meriandra dianthera* showed the highest levels of Mn, Cr, V, and other elements and the values were significantly different (*P* < 0.05).

## 1. Introduction

Trace elements have been identified for a long time as potential candidates for improving metabolic disorders including diabetes [1]. It is widely believed that some trace elements, such as Zn, Cr, V, Mg, Mn, and Se serve as cofactors of antioxidative enzymes and play an important role in protecting the insulin secreting pancreatic *β*-cells, which are sensitive to free radical damage [2, 3]. It has also been reported that the imbalance of some essential trace elements might adversely affect pancreatic islet and cause development of diabetes [4] and thus some trace elements have been recommended as dietary supplement to alleviate the impaired insulin metabolism in diabetic patients [5, 6]. Some researchers have also shown that trace elements beneficially affect the complications of diabetes mellitus [7]. Clinical studies suggest that the body's balance of mineral trace elements is disrupted by diabetes and thus diabetic individuals are susceptible to trace element deficiency [8]. Even though trace elements are important for the normal functioning of the body, they can be harmful and toxic at high concentrations [9]. Therefore profiling the levels of these elements is mandatory in monitoring the safety of herbal preparations employed in the management of diabetes and other ailments.

The most widely and commonly used techniques of elemental analysis, providing acceptable levels of precision and accuracy, include flame atomic absorption spectrometry (FAAS), graphite furnace atomic absorption spectrometry (GFAAS), inductively coupled plasma atomic emission spectrometry (ICP-AES), and inductively coupled plasma mass spectrometry (ICP-MS) [10]. The present work focuses on analysis of the levels of trace elements and their therapeutic role in the management of diabetes from selected Eritrean medicinal plants. The investigated plants were* Aloe camperi*,* Meriandra dianthera*,* Lepidium sativum*,* Brassica nigra*, and* Nigella sativa*. These plants have been traditionally used for the treatment of diabetes and other ailments in Eritrea [11]. The profile of trace and other elements in* Aloe camperi* and* Meriandra dianthera* is reported for the first time in this paper.

## 2. Materials and Methods

### 2.1. Sampling

*Aloe camperi and Meriandra dianthera* are wild plants and their leaves were collected from Adi-Hawisha (15°14′34′′ North 38°58′14′′ East), Central Zone, Eritrea. Moreover, dry seeds of* Lepidium sativum*,* Brassica nigra*, and* Nigella sativa* were purchased from a local market in Asmara, Eritrea. The plants were washed using distilled water, dried in shade, and then heated on a heating block in an oven for complete dryness. They were finely powdered using an electric blender and fed through a sieve (0.50 mm) and saved until further use.

### 2.2. Chemicals and Reagents

Analytical grade chemicals and reagents were purchased from Sigma-Aldrich Company. 65% nitric acid (HNO_3_), 30% hydrogen peroxide (H_2_O_2_), 32% Hydro chloric acid (HCl), and 98% sulphuric acid (H_2_SO_4_) were used for digestion purposes. Ultrapure-deionized water (18 Ω) was used throughout the study. The glassware was soaked in 3 M HNO_3_ for the whole night and washed and rinsed with deionized water to minimize the chances of interferences. All the chemical analyses were conducted under extractor hood and a digital IR Vortex Mixer (S/N296058 made in Italy) was used for mixing of the solutions.

### 2.3. Standards and Calibration

Different custom-grade multielement standard solutions of PerkinElmer Pure were employed for the calibration and in-house certified reference materials (CRMs) for biological materials, developed using the “ISO Guide 80,” were used for comparison purposes [14]. Calibration curves for each element were constructed in triplicate using six different concentrations, giving regression coefficient (*r* ^2^) values which ranged from 0.9957 to 0.9996. An internal-standard stock solution of 100 mg/L Lutetium (Lu) was prepared from single-element stock solutions.

### 2.4. Sample Digestion and Preparation

Each sample (0.5 g) was weighed accurately using a 4-decimal-place analytical balance and placed in a digestive tube. The samples were initially digested with concentrated HNO_3_ (5 mL) at 175°C for 40 min and then at 150°C for 90 min. After the mixture was cooled, 1 mL HNO_3_ and 0.5 mL H_2_SO_4_ were added and the mixture was heated at 175°C for 60 min. The mixture was allowed to cool and H_2_O_2_ (2 mL) was added dropwise and heated at 140°C for 10 min to remove any remaining NO_2_ that might interfere in the measurement. The resulting mixture was transferred to a calibrated flask and HCl (8 mL) was added and the entire filtrate was diluted suitably with ultrapure deionized water to 25 mL. Samples were immediately analysed following the digestion.

### 2.5. Instrumental Analysis

A dual viewing ICP-OES (Perkin Elmer Optima 8300, made in Singapore) coupled to an ultrasonic nebulizer CETAC 6000AT+ (CETAC, Omaha, NE, USA) was employed for the analysis of the trace and other elements. The Windows 7 compatible S/W provided by Perkin Elmer was used to process the spectral data for calculating sample concentrations by comparing light intensities measured at various wavelengths for standard solutions with intensities from the sample solutions. The operating conditions set for the ICP-OES are shown in Table 1. Moreover, the levels of Cu, Pb, Zn, and Fe were studied using FAAS (Agilent 240FS, made in Australia). The dilute filtrate solutions of the digested plant samples were transferred into test tubes and about 20 mL of the resulting solution was then fed in to the air-acetylene FAAS. Suitable hollow cathode lamps were used to measure the absorbance of the elements at their resonance wavelengths. The optimized operating conditions for the FAAS are shown in Table 2.

### 2.6. Statistical Analysis

Data for the concentrations of trace and other elements were processed to obtain mean and standard error of mean (SEM). One-way analysis of variance followed by Student's *t*-test was used to compare the mean values. A value of *P* < 0.05 was considered to be statistically significant.

## 3. Results and Discussion

**Figure 1:**
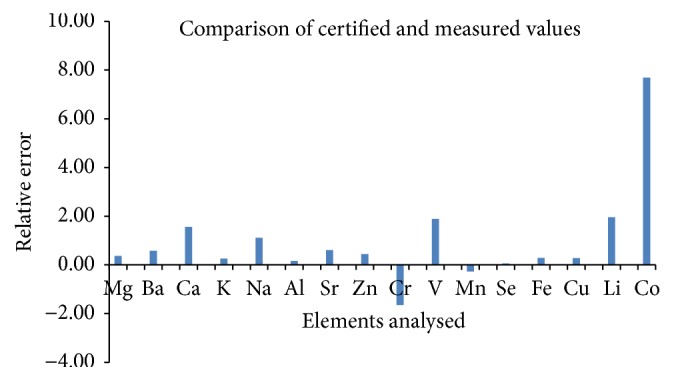
The relative error based on comparison of the certified and measured values.

Before the analysis of the elements, the accuracy of the methods was verified using in-house certified reference materials (CRMs) digested using dry ashing. As exhibited in Figure 1, the calculated relative errors were as follows: Mg: 0.36, Ba: 0.58, Ca: 1.56, K: 0.26, Na: 1.11, Al: 0.17, Sr: 0.61, Zn: 0.44, Cr: −1.65, V: 1.89, Mn: −0.27, Se: 0.06, Fe: 0.28, Cu: 0.28, Li: 1.96, and Co: 7.69. Except for Cr and Mn, all the elements demonstrated positive relative errors and the deviations from the mean values were small. There was no significant difference in the measured and certified values. Therefore, the calculated relative errors revealed high accuracy of the method, suggesting that this method can be used for routine analyses of trace and heavy metals in herbal products. The concentrations of the elements analysed using the ICP-OES and FAAS are furnished in Tables 3(a) and 3(b). A total of 16 elements were analysed and special emphasis was given to the levels of Zn, Cr, Se, Mg, Mn, V, and Mg. Summary of the results and related discussions of the trace elements and magnesium are briefly described below.

### 3.1. Zinc (Zn)

In this study, a substantial quantity of Zn was determined in all the plants. Maximum amount (in ppm, dry weight) of Zn was found in the seed samples of* Nigella sativa* (52.23), followed by* Brassica nigra* (37.90), and the lowest in* Aloe camperi* (23.25). Based on Tukey's multiple comparisons test, there was significant difference (*P* < 0.01) in the levels of Zn among the plants. Zn plays a crucial role in the storage and secretion of insulin, which subsequently increases the uptake of glucose [15]. The role of Zn in the production of insulin and catalysis of numerous enzymatic reactions has been reported [16]. The low level of Zn in plasma adversely affects the ability of islet cells to produce and secrete insulin [17]. Moreover, it may aggravate the insulin resistance in type 2 diabetes and thus causes other complications [18].

### 3.2. Chromium (Cr)

Cr was found in the range of 1.18 to 1.86 ppm, where the highest concentration was detected in* Meriandra dianthera*, while the lowest value was in* Nigella sativa*. There was no significant difference in the levels of Cr among the plants. Cr is a crucial trace element with many sites of action and has a vital biological activity which is necessary in glucose homeostasis [19]. It regulates insulin and blood glucose levels by stimulating insulin signalling pathway and metabolism and thus it may improve insulin sensitivity. Modulation of lipid metabolism by Cr in peripheral tissues may represent an additional novel mechanism of action [20]. Deficiency of Cr or its biological active form has been implicated in the pathogenesis of insulin resistance and diabetes [8].

### 3.3. Vanadium (V)

The concentration (in ppm) of V in the medicinal plants ranged from 1.05 in* Lepidium sativum* to 9.38 in* Meriandra dianthera*. The levels of V were significantly different (*P* < 0.01) among the plants. Vanadium has been known for long to possess antidiabetic properties [21]. It affects various aspects of carbohydrate metabolism including glucose transport, glycolysis, and glucose oxidation and glycogen synthesis [22]. Vanadium acts primarily as an insulin mimetic agent, although enhanced insulin activity and increased insulin sensitivity have also been noted [23]. Besides, it stimulates glucose uptake without affecting endogenous levels [24].* In vivo* studies show the beneficial effects of various vanadium salts in mild or severe diabetes [22].

### 3.4. Manganese (Mn)

Based on the results portrayed in Table 3(b), the lowest concentration of Mn was observed in* Lepidium sativum* and amounted to 18.51 ppm, while the highest level (82.03 ppm) was in* Meriandra dianthera*. The level of Mn, among the plants, was significantly different (*P* < 0.001). It has been reported that Mn is involved in normal immune functions, regulation of blood sugar and cellular energy, and the defence mechanisms against free radicals [25]. Mn activates certain enzymes that play important roles in the metabolism of carbohydrates, amino acids, and cholesterol. Mn is also required for normal insulin synthesis, its secretion, and an alteration in its metabolism has been implicated in diabetes development [26]. Mn deficiency can result in impaired glucose tolerance, altered carbohydrate and lipid metabolism, impaired insulin secretion, and skeletal abnormalities [27].

### 3.5. Selenium (Se)

Investigation of the level of Se revealed that the element exists in a very low concentration in all the plants. The range of Se was 25.47 up to 72.64 ppb whereby the highest level of the element was observed in* Nigella sativa*. The values of Se were not statistically different among the investigated plants. Due to its antioxidant properties, Se might be able to prevent the development of diabetes and associated complications [23]. In addition, selenate, an inorganic form of Se, mimics insulin activity in experimental models [28]. It has been reported that Se could also affect carbohydrate metabolism and the blood glucose-lowering action of oral selenate has been demonstrated in chronically treated diabetic rats [29].

### 3.6. Magnesium (Mg)

The level of Mg was detected in substantial quantity in all the plants. It ranged from 10.8 ppm in* Aloe camperi* to 25.05 ppm in* Nigella sativa*. The level of Mg was relatively variable and thus was statistically different (*P* < 0.01). Mg is a cofactor of various enzymes in carbohydrate oxidation and plays an important role in glucose transporting mechanism of the cell membrane. It is also involved in insulin secretion, binding, and activity [30]. It has a vital role in the phosphorylation reactions of glucose and its metabolism and it may influence the release and activity of the hormones that help control blood glucose levels [31]. It was demonstrated that Mg deficiency might lead to a decrease in insulin mediated glucose uptake and has been associated with the development of insulin resistance [32].

**Figure 2:**
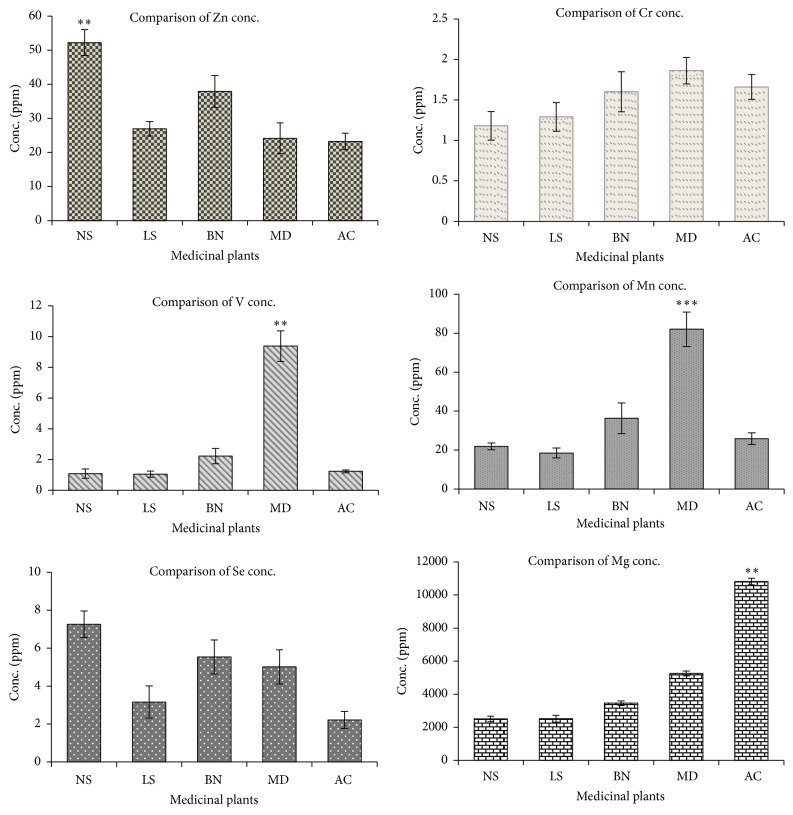
Comparison of the levels of Zn, Cr, V, Mg, Mn, and Se among the plants. ^*∗∗∗*^ *P* < 0.001 and ^*∗∗*^ *P* < 0.01 represent statistical significance of the level of the element in one plant relative to the others.

As presented in Figure 2,* Meriandra dianthera* showed higher levels (in ppm) of the trace elements including Mn, V, and Cr (82.03, 9.38, and 1.86, resp.). The plant also exhibited the highest levels (in ppm) of Fe (1241.05) and Co (1.48) as compared to the other plants. Iron is an important trace element and iron protein mixtures play vital role in the metabolism of all living organisms [33] and cobalt has been demonstrated to boost the effects of insulin and its action and the efficiency of cobalt as an antidiabetic agent has been proven [34]. As recently published, the* in vivo* antidiabetic activity of* Meriandra dianthera* elicited the highest glucose-lowering effect [35] compared to the other plants and thus the reported bioactivity of the plant may be partly attributed to the presence of some of the trace elements that are involved in insulin production and action.

Furthermore,* Meriandra dianthera* offered the highest level of the major elements including Ca, Al, and Li. Special mention should be made of Ca and Al, which have anomalous values (in ppm) of 7341.51 and 2810.91, respectively. These values are extremely higher compared to the values measured in the other plants (*P* < 0.001). Similarly, as shown in Table 3(a),* Aloe camperi* displayed the highest levels (in ppm) of Mg, K, and Sr (10811, 10646, and 169, resp.) compared to the other plants (*P* < 0.01). However, the plant showed lowest levels of Zn, V, and Se compared to the other plants (Figure 1). There are no reported levels of trace and major elements related to* Meriandra dianthera* and* Aloe camperi.*

The results found in the present work were compared with the values previously reported and thus analysis of* Nigella sativa* seeds from Turkey showed that they have 0.12, 117.32, 41.42, 30.26, 28.56, and 2.55 ppm of Co, Fe, Zn, Cu, Mn, and Cr, respectively [36]. Apart from the levels of Fe and Co, all the values found in this study were similar to the levels of the elements reported from Turkey. Moreover, Shomar [37] reported the levels of Cr, Cu, Fe, Mn, Al, Ba, Mg, and K in* Nigella sativa* from Egypt as 4.2, 14.5, 114, 47.4, 99.2, 14.0, 2356, and 9900 ppm, respectively. Current results presented elevated levels of Cu, Fe, and Al compared to the values reported from Egypt. On contrary, the levels of Mn, Cr, Ba, and K reported from Egypt were higher than the values determined in this paper. Similarly, analysis of the levels (in ppm, dry weight) of* Brassica nigra* seeds from India using AAS [38] gave lower levels of Cu (5.50) and Fe (184.0) compared to the current report of the elements (32.97 and 847.64, resp.). However, the levels of Zn and Mn were very similar in both reports. Plants have the ability to uptake metals as nutrient from the soil and its environment which are so essential for their physiological and biochemical growth and thus the difference in the levels of the trace elements from different countries could be due to the variation in soil types, agricultural and industrial activities, and local growing conditions such as differences in water, plant interactions, and weather [39, 40].

The highest levels of Cu and Se were observed in the seeds of* Nigella sativa* (36.21 ppm and 72.64 ppb, resp.). Low concentrations of Co (0.19–1.48 ppm) and Li (0.18–0.86 ppm) were observed in all the plant samples. Besides, the concentrations of metals such as lead (Pb), cadmium (Cd), and arsenic (As) present in the leaves and seeds of the plants were not quantifiable. It is known that excess concentration of these elements is toxic and thus can disrupt the glucose uptake and alter the related molecular mechanism in glucose regulation [41]. Based on literature review [12, 13], Tables 3(a) and 3(b) summarized the recommended dietary allowance (RDA) and tolerable upper levels (UL) of most of the elements and thus it can be deduced that the existing profile of the trace and other elements, based on WHO 2008 and WHO 2011 reports [42, 43], was within the permissible limits.

## 4. Conclusion

Overall, the results indicated the presence of characteristic level of trace elements including Zn, Cr, V, Mn, Se, and Mg that are associated with the glucose-lowering effects. The levels of the trace elements were within the permissible limit set by the FAO/WHO. Even though the relationship between diabetes and trace elements is complex and is most probably related to a combination of multiple interacting effects, the results obtained can be supplementary to the reported bioactivities of the medicinal plants.

## Figures and Tables

**Table 1:** The operating conditions of the ICP-OES.

Condition	Setting
Power	1.3 kW
Plasma gas glow	15 L/min
Auxiliary gas flow	1.5 L/min
Spray chamber type	Glass cyclonic (single-pass)
Torch	Standard one-piece quartz axial
Nebulizer type	Sea spray
Nebulizer flow	0.7 L/min
Pump speed	2–4 rpm
Total sample usage	2 mL
Replicate read time	5 s
Number of replicates	2
Sample undertake delay time	15 s
Stabilization time	40 s
Rinse time	20 s
Fast pump	Off
Background correction	Fitted

**Table 2:** The operating conditions of the FAAS.

Parameter	Element matrix			
	Cu	Pb	Zn	Fe
Wavelength	324.8 nm	283.3 nm	213.9 nm	372.0 nm
Slit width	0.5 nm	0.5 nm	0.5 nm	0.2 nm
Gain	60%	29%	33%	55%
Lamp current	4.0 mA	10.0 mA	5.0 mA	5.0 mA
Flame type	Air/acetylene	Air/acetylene	Air/acetylene	Air/acetylene
Air flow	13.50 L/min	13.50 L/min	13.50 L/min	14.90 L/min
Acetylene flow	2.20 L/min	2.20 L/min	2.50 L/min	2.20 L/min

**(a):** 

Samples	Mg	Ba	Ca	K	Na	Al	Sr
NS	2505.52 ± 11.43	36.21 ± 1.08	478.35 ± 5.99	2496.26 ± 11.43	21.84 ± 0.22	126.71 ± 4.73	92.97 ± 0.81
LS	2516.46 ± 13.62	31.77 ± 4.99	497.27 ± 4.41	2464.39 ± 13.62	18.51 ± 0.32	131.90 ± 1.67	15.50 ± 0.68
BN	3460.00 ± 9.12	32.97 ± 3.30	1406.30 ± 3.04	3388.48 ± 9.12	36.31 ± 0.98	446.42 ± 5.16	53.89 ± 1.60
MD	5263.06 ± 9.49	28.61 ± 3.51	7341.51 ± 4.05^*∗∗∗*^	5219.54 ± 9.49	82.03 ± 1.10^*∗∗∗*^	2810.91 ± 7.08^*∗∗∗*^	82.12 ± 0.80
AC	10811.66 ± 13.13^*∗∗*^	18.41 ± 1.00	545.32 ± 7.96	10655.91 ± 9.13^*∗∗∗*^	25.78 ± 0.38	196.79 ± 4.13	169.51 ± 2.95^*∗∗∗*^

RDA	280–350 mg	1.1 mg	1000 mg	3.5 g	1.5 g	—	—
UL	350 mg	—	2500 mg	3000 mg	2300 mg	—	—

NS: *Nigella sativa*, LS: *Lepidium sativum*, BN: *Brassica nigra*, AC: *Aloe camperi*, and MD: *Meriandra dianthera*.

RDA: recommended daily dietary allowance per day for adults; UL: tolerable upper intake level per day for adults [12, 13].

Concentration values are expressed as mean ± SD (*n* = 3); ^*∗∗∗*^ *P* < 0.001 and ^*∗∗*^ *P* < 0.01 (statistical significance compared to all the other plants).

**(b):** 

Samples	Zn	Cr	V	Mn	Se^#^	Fe	Cu	Li	Co
NS	52.23 ± 0.95^*∗∗*^	1.18 ± 0.04	1.08 ± 0.03	21.84 ± 0.22	72.64 ± 2.98^NS^	478.35 ± 5.99	36.21 ± 1.08^NS^	0.52 ± 0.05	0.28 ± 0.02
LS	26.94 ± 1.53	1.29 ± 0.05	1.05 ± 0.02	18.51 ± 0.32	33.64 ± 5.47	497.27 ± 4.41	31.77 ± 4.99	0.23 ± 0.02	0.19 ± 0.03
BN	37.90 ± 1.84	1.60 ± 0.04	2.23 ± 0.05	36.31 ± 0.98	58.73 ± 3.92	847.64 ± 3.09	32.97 ± 3.30	0.18 ± 0.06	0.29 ± 0.01
MD	24.17 ± 1.10	1.86 ± 0.03^NS^	9.38 ± 0.10^*∗∗*^	82.03 ± 1.10^*∗∗∗*^	46.81 ± 5.96	1241.05 ± 3.17^*∗∗∗*^	28.61 ± 3.51	0.86 ± 0.10^*∗*^	1.48 ± 0.08^*∗∗∗*^
AC	23.25 ± 0.63	1.66 ± 0.01	1.23 ± 0.01	25.78 ± 0.38	25.47 ± 3.66	545.32 ± 7.96	18.41 ± 1.00	0.39 ± 0.05	0.25 ± 0.04

RDA	7–9 mg	25–35 *µ*g	10–20 *µ*g	3 mg	50–60 mcg	9–15 mg	900 *µ*g	—	—
UL	25 mg	1000 mcg	1.8 mg	11 mg	300 mcg	25 mg	5 mg	—	—

NS: *Nigella sativa*, LS: *Lepidium sativum*, BN: *Brassica nigra*, AC: *Aloe camperi*, and MD: *Meriandra dianthera*.

RDA: recommended daily dietary allowance per day for adults; UL: tolerable upper intake level per day for adults [12, 13].

Concentration values are expressed as mean ± SD (*n* = 3); ^#^concentration of Se expressed as ppb (*µ*g·kg^−1^).

^*∗∗∗*^ *P* < 0.001, ^*∗∗*^ *P* < 0.01, and ^*∗*^ *P* < 0.05; NS: not significant (statistical significance compared to all the other plants).
